# Impact of combined exercise training in peripheral and diaphragm muscles and in mortality in a preclinical model of pulmonary arterial hypertension

**DOI:** 10.1007/s00424-025-03118-z

**Published:** 2025-09-18

**Authors:** Thais C. Freire, Antônio V. Nascimento-Filho, Marília S. Ferreira, Danielle da Silva Dias, Victor Miranda, Marina Dutra, Larissa Seibt, Andrey Serra, Denielli da Silva Gonçalves Bos, Maria Cláudia Irigoyen, Marcelle Paula-Ribeiro, Kátia De Angelis

**Affiliations:** 1 Translational Physiology Laboratory, Universidade Nove de Julho (UNINOVE), São Paulo, Brazil; 2 Exercise Physiology Laboratory, Department of Physiology, Federal University of Sao Paulo (UNIFESP), Botucatu Street 862, Biomedical Sciences Building, 5Th Floor, Sao Paulo, 04023-062 Brazil; 3 Postgraduate Program in Physical Education, Federal University of Maranhão, São Luís, Brazil; 4 Laboratory of Physiology and Cardiac Pathophysiology, Department of Medicine, UNIFESP, Sao Paulo, Brazil; 5 Department of Physical Therapy, FMUSP, Sao Paulo, Brazil; 6 Hypertension Unit, InCor, FMUSP, São Paulo, Brazil; 7 School of Medicine and Population Health, University of Sheffield, Sheffield, UK

**Keywords:** Pulmonary arterial hypertension, Oxidative stress, Inflammation, Exercise training

## Abstract

Pulmonary arterial hypertension (PAH) is a progressive disease characterised by systemic oxidative stress and inflammation that extends beyond the pulmonary vasculature to the musculoskeletal system. Combined exercise training (ET), incorporating aerobic and resistance components, is a promising non-pharmacological intervention, but its effects on musculoskeletal oxidative stress and inflammation remain unclear. To evaluate the effects of combined ET on musculoskeletal oxidative stress and inflammation, muscle wasting, and survival in a monocrotaline (MCT)-induced PAH. Male Wistar rats were assigned to MCT-treated sedentary (MCT-SED) or ET (MCT-ET) groups (*n* = 12/group), or saline-treated sedentary (SAL-SED) or ET (SAL-ET) controls (*n* = 8/group). PAH was induced via MCT injection (MCT, 40 mg/kg). The ET consisted of moderate-intensity interval aerobic (3x/week) and resistance (2x/week) training for four weeks. Muscle mass, oxidative stress and inflammation markers (IL-6, IL-10, TNF-α) were assessed in gastrocnemius and diaphragm muscles. PAH increased oxidative damage, reduced antioxidant defences, and elevated inflammatory markers in both muscles, contributing to muscle loss. Combined ET enhanced gastrocnemius antioxidant capacity (FRAP, SOD), reduced pro-oxidants (hydrogen peroxide, nitrite), and attenuated oxidative damage (TBARS, carbonyls) in both muscles. Combined ET decreased pro-inflammatory markers (IL-6, TNF-α), prevented diaphragm atrophy, and improved survival (MCT-SED vs. MCT-ET, *p* = 0.03; hazard ratio, 4.3; 95% CI, 1.2–15.1). Combined interval ET improved redox balance and inflammatory profiles in both peripheral and respiratory muscles. These adaptations were linked to reduced diaphragm muscle wasting and enhanced survival in MCT-induced PAH. Our findings support combined ET as a non-pharmacological strategy for managing systemic complications of PAH.

## Introduction

Pulmonary arterial hypertension (PAH) is a progressive disease characterised by increased pulmonary vascular resistance, leading to right ventricular failure and, ultimately, death [26, 30, 38]. While the primary pathology involves the lungs and heart [12, 15], alterations in function and structure beyond these organs—particularly in the musculoskeletal system [11, 41] —have been increasingly recognised as contributors to disease progression and poor clinical outcomes [8, 43]. Peripheral skeletal muscle dysfunction [48] and impaired respiratory muscle performance [52], which together result in reduced functional capacity [48, 52], are commonly observed in PAH patients, further emphasising the systemic nature of the disease [28].

Oxidative stress and inflammation are critical mediators of PAH progression and poor prognosis [4, 10, 21, 28]. Oxidative stress arises from an imbalance between reactive oxygen species (ROS) production and antioxidant defences, leading to cellular damage and tissue dysfunction [56, 58]. Simultaneously, elevated levels of pro-inflammatory cytokines intensify vascular and systemic inflammation, further exacerbating disease pathology [18, 19]. These interconnected processes not only accelerate pulmonary vascular remodeling but also contribute to dysfunction in peripheral and respiratory skeletal muscles [59, 60]. Emerging evidence suggests that heightened inflammatory signaling and excessive ROS production play a central role in PAH-related myopathy and muscle wasting, perpetuating chronic fatigue and significantly reducing quality of life (QoL) in PAH patients [8, 10, 14, 53, 56, 60].

Conversely, exercise training (ET) has emerged as a non-pharmacological strategy to mitigate these adverse outcomes [42, 46, 68]. Growing evidence has established ET as an adjuvant component of PAH management [32, 33], demonstrating safety and efficacy in improving exercise capacity, QoL, and even survival [32, 33]. Mechanistically, both aerobic [16, 17, 64] and resistance ET provide significant benefits [63]. When performed in isolation, these modalities increase exercise tolerance, prevent pathological remodelling, and improve right heart function and oxidative stress markers [13, 16, 17, 63, 64]. However, in clinical practice, the most common approach is multimodal, combining aerobic and resistance training, particularly for stable PAH patients [32, 34]. Additionally, interval training, which alternates periods of moderate/high-intensity exercise with low-intensity recovery [13], has gained popularity due to its ability to enhance exercise performance while minimizing cardiocirculatory strain [6, 69].

Despite its widespread clinical acceptance, the effects of multimodal and interval ET on oxidative stress and inflammation remain underexplored [44, 45], particularly regarding systemic impacts beyond the lungs and heart. This study, therefore, utilised an MCT-induced PAH model to investigate the effects of combined and interval ET on oxidative stress and inflammation in skeletal and respiratory muscles after the establishment of the disease. Furthermore, we examined its impact on muscle mass, as an indicator of muscle wasting, and its influence on survival. Understanding these mechanisms is crucial for optimising exercise-based interventions and addressing the broader systemic dysfunctions that contribute to disease progression.

## Methods

### Methods

#### Experimental design and induction of PAH

Male Wistar rats (*n* = 40, 250–300 g) were obtained and housed at 22 °C under a 12-h dark/light cycle, with ad libitum access to water and standard rodent chow. All procedures involving experimental animals were approved by the Institutional Animal Care and Use Committee (CEUA–UNIFESP; registration number: 2020/5905261120). On day 1, animals were randomly allocated into four groups: MCT-SED (PAH-induced; sedentary), SAL-SED (control; sedentary), MCT-ET (PAH-induced; exercise-trained), and SAL-ET (control; exercise-trained). PAH was induced in the experimental groups via a single subcutaneous injection of monocrotaline (MCT, 40 mg/kg of body weight), while control groups received an equivalent volume of saline [36]. This dose was previously demonstrated to mimic a stable PAH with preserved cardiac output.

Two weeks after MCT or saline administration, transthoracic echocardiography was performed on moderately anaesthetised animals (3% isoflurane in 100% oxygen for induction and 1.5% for maintenance) by an independent researcher blinded to the experimental groups. Images were captured in accordance with the recommendations of the American Society of Echocardiography and subsequently stored for analysis [61]. PAH was confirmed using markers of increased pulmonary vascular resistance and mean pulmonary arterial pressure (mPAP), including a decreased pulmonary artery acceleration time (PAAT)-to-right ventricular ejection time ratio (PAAT/EjT), an increased pulmonary artery-to-aorta diameter ratio (PA/Ao ratio), and an estimated mPAP [calculated using the formula 58.7 – (1.21 × PAAT)] [67]. These measurements were repeated after four weeks, at the end of the experimental timeline.

#### Exercise training protocol

Seven days after MCT or saline injection, all animals began familiarisation with treadmill running (10 min/day at 0.3 km/h) [27] and climbing exercises (54 vertical steps, each 0.5 cm in height/step) [62] over a period of five days.

Following this familiarisation period, the animals underwent maximal exercise tests on a motorised treadmill (Imbramed TK-01, Brazil) and a ladder to determine baseline parameters for aerobic capacity (maximum running speed) and resistance capacity (maximum load), respectively. These baseline values were subsequently used to prescribe the ET programme.

During the maximal running test, treadmill speed was increased incrementally by 3 m/min every three minutes until exhaustion. The maximum speed was defined as the highest speed completed by the animal before reaching exhaustion [27]. The dynamic resistance exercise test involved an initial load equivalent to 75% of the animal's body weight. After a two-minute rest period, an additional 15% of body weight was added for each subsequent climb until exhaustion, as previously described by Sanches et al. [62].

The combined ET protocol began after confirmation of PAH/control status (14th day post-injection) and was carried out over a four-week period, with progressively increasing duration and intensity. Both modalities aimed to replicate evidence-based recommendations for exercise in PAH [33, 35].

The sessions alternated aerobic ET on a treadmill (three times per week) with dynamic resistance ET on the ladder (twice per week). The aerobic sessions consisted of moderate-intensity interval training, with four-minute intervals at 60% of the maximum running speed, followed by two-minute recovery intervals at 40%, for a total session duration of approximately 30 to 40 min [1]. The resistance ET was performed at 40–60% of the maximum load, with each session lasting approximately 20–30 min and consisting of 15–20 climbing repetitions, with a one-minute rest interval between repetitions [27, 62]. Throughout these interventions, the SED groups were kept in the same laboratory as the ET groups to ensure they were exposed to identical environmental conditions.

### Tissue preparation

At the conclusion of the experimental procedures (day 47 post-MCT/saline injection), all groups were euthanised via decapitation. The gastrocnemius and diaphragm muscles were dissected, weighed, and muscle weight was used as a proxy for muscle wasting. Subsequently, these tissues were flash-frozen at –80 °C for further analysis.

### Inflammatory mediators

The concentrations of interleukins (IL-6 and IL-10) and TNF-α in gastrocnemius and diaphragm homogenates were quantified using specific ELISA kits (R&D Systems Inc.), as previously described [27].

### Oxidative stress markers

The methodologies for evaluating oxidative stress profiles have been detailed in previous studies [25, 27, 55]. Protein quantification was performed using the method described by Lowry et al. [47].

The antioxidant assay to determine the ferric reducing antioxidant power (FRAP) was conducted in a microplate format. A mixture of 290 μL of FRAP reagent (composed of sodium acetate buffer and acetic acid, pH 3.6; 10 mM TPTZ; and 20 mM ferric chloride hexahydrate) and 10 μL of either a ferrous sulphate heptahydrate standard solution or gastrocnemius and diaphragm muscle homogenates was prepared. The microplate was incubated with shaking at 37 °C for five minutes and reading at 593 nm in IL-593 spectrophotometer. Results are expressed in μmol/mg protein [9].

Superoxide dismutase (SOD) activity was quantified based on the inhibition of the reaction between O₂˙ − and pyrogallol. The assay consisted of 980 μL of 50 mM Tris-base buffer containing 1 mM EDTA, 5 μL of catalase (CAT), 5 μL of homogenate, and 10 μL of 24 mM pyrogallol. Absorbance kinetics were monitored for 120 s at 420 nm using an IL-593 spectrophotometer [51]. The CAT activity was determined by measuring the decrease in hydrogen peroxide (H₂O₂) absorbance at 240 nm. The assay consisted of 980 μL of 50 mM phosphate buffer, 10 μL of homogenate, and 10 μL of H₂O₂. The decrease in absorbance kinetics was monitored for 60 s using an IL-593 spectrophotometer [3].

The H₂O₂ concentration was assessed via phenol red oxidation mediated by horseradish peroxidase (HRPO) in the presence of H₂O₂. The assay was conducted using 70 μL of homogenate and 180 μL of HRPO buffer (dextrose buffer + phenol red + HRPO), incubated at 25 °C for 25 min. After incubation, 5 μL of sodium hydroxide was added to stop the reaction. Absorbance was measured at 630 nm using a microplate reader [57].

The activity of NADPH oxidase was evaluated in homogenates of peripheral and diaphragm muscles by superoxide production. The assay was conducted using 170 μL of 50 mM phosphate buffer containing 2 mM EDTA and 150 mM sucrose, 20 μL of 1.3 mM NADPH, and 10 μL of homogenate. Kinetic absorbance was measured at 340 nm using a microplate reader [66].

Lipid peroxidation was assessed by measuring thiobarbituric acid-reactive substances (TBARS), with results expressed as nmol/mg protein, following the adapted method of Ohkawa et al_*.*_ [54]. Protein oxidation was determined using the carbonyl detection technique and absorbance was measured at 535 nm in an IL-593 spectrophotometer [55].

Nitrite (NO₂⁻) levels were determined using the Griess reagent, which forms a chromophore with strong absorbance at 540 nm upon reaction with a mixture of naphthyl ethylenediamine (0.1%) and sulfanilamide (1%). A standard curve was established using a series of serial dilutions (10⁻⁸–10⁻^3^ mol/L) of sodium nitrite [27].

### Statistical analysis

Sample size was based on previous studies of exercise effects in MCT-induced PAH models [13, 36] requiring a minimum of 8 animals per group. To account for an expected 30%−40% mortality associated to MCT injection [22], 4 additional animals were included in each MCT group (12 animals/group). Data are presented as mean ± standard deviation. Normality and homogeneity assumptions were tested using the Shapiro–Wilk and Levene tests, respectively.

Group comparisons were performed using two-way analysis of variance (ANOVA), followed by Tukey’s post hoc test. Partial eta squared (η^2^ₚ) was used to report effect sizes for main effects when no significant interaction was present. When a significant interaction was detected, Cohen’s d was calculated for relevant pairwise comparisons. Effect sizes were interpreted using standard benchmarks: small (η^2^ₚ ≈ 0.01; d = 0.2), medium (η^2^ₚ ≈ 0.06; d = 0.5), and large (η^2^ₚ ≈ 0.14; d = 0.8) [29]. Pearson’s correlation coefficient (r) was calculated to assess the relationship between muscle weight and markers of inflammation and oxidative stress. Survival analysis for the MCT groups (e.g., SED vs. ET) was conducted using Kaplan–Meier curves, followed by the log-rank test. A significance level of 5% was adopted for all statistical analyses.

## Results

### Indicators of PAH Establishment and effect of combined ET

**Fig. 1:**
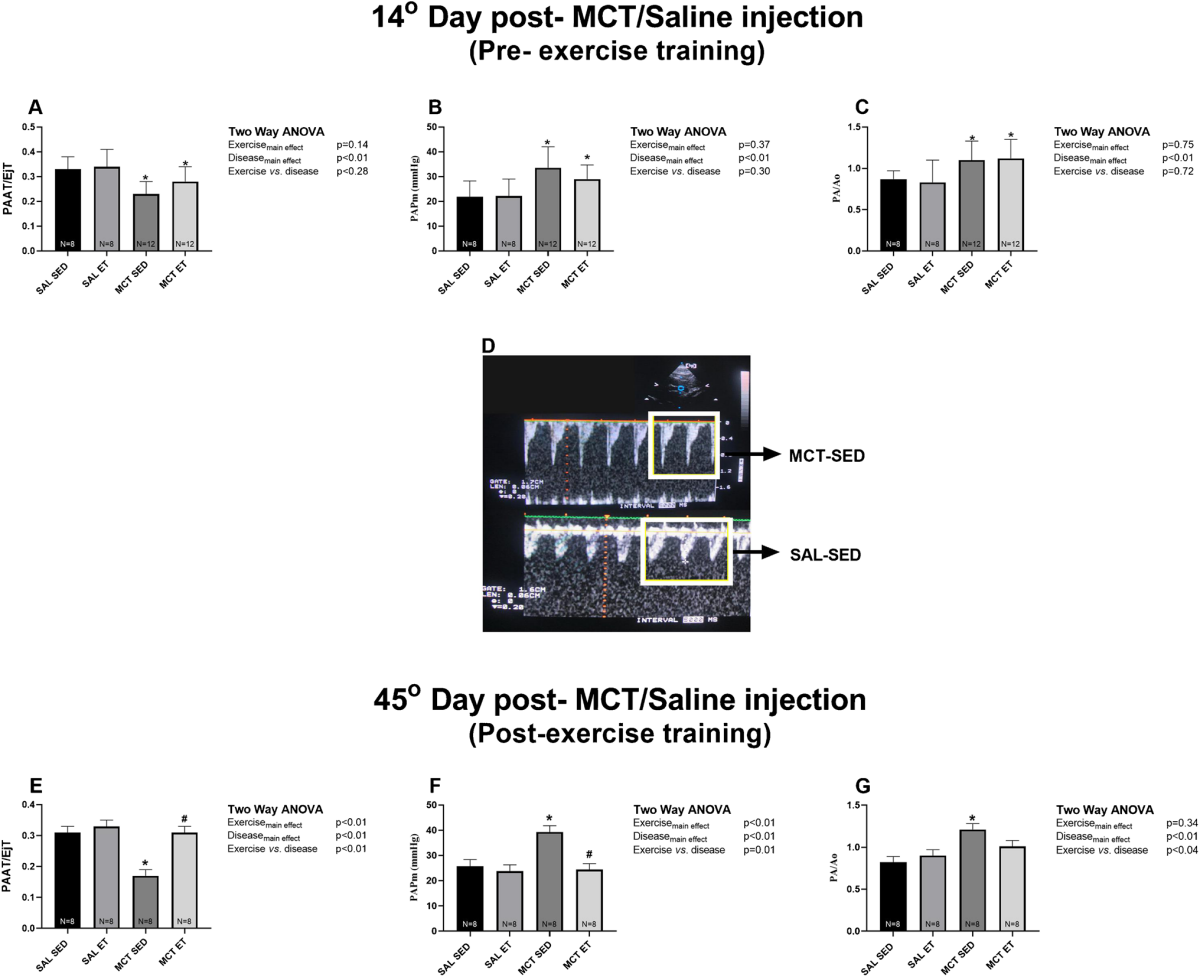
Echocardiographic assessments in monocrotaline (MCT)-induced pulmonary arterial hypertension (PAH) and controls (SAL). SED: sedentary; ET: exercise training. PAAT/EjT: pulmonary artery acceleration time (PAAT)-to-right ventricular ejection time ratio; PA/Ao ratio: increased pulmonary artery-to-aorta diameter ratio; mPAP: estimated mean pulmonary arterial pressure. Panels **A-C**: pre-ET; Panels **E–G**: post-ET; Panel **D**: Representative echocardiographic images of MCT-SED and SAL-SED groups. Data are expressed as mean ± SD. *p < 0.05 vs. SAL, #*p* < 0.05 vs. SED. Please refer to Panels **E–G**. One animal from the MCT-ET group is absent due to the inability to obtain echocardiographic measurements, as the animal exhibited resistance to anaesthesia

Echocardiographic analysis revealed a reduced PAAT/EjT ratio and an increased PA/Ao ratio in the MCT groups compared to the SAL groups (P_disease_ =  < 0.01; Fig. 1A and C, respectively), indicating elevated pulmonary vascular resistance. The MCT groups also exhibited an increased mPAP compared to the SAL groups (P_disease_ =  < 0.01; Fig. 1B). A midsystolic notch in the pulmonary artery flow wave, indicative of downstream vascular remodelling and stiffening, was observed in all MCT groups (Fig. 1D, upper quadrant).

A second echocardiographic assessment, conducted after the four-week ET period, revealed that these alterations persisted in the MCT-SED group (Fig. 1E–G). However, combined ET effectively reversed these changes in the PAH group, as indicated by significant improvements in PAAT/EjT (*P* < 0.01; Cohen’s *d* = 5.29; MCT-ET vs. MCT-SED) and mPAP (*P* < 0.01; Cohen’s *d* = 4.45; MCT-ET vs. MCT-SED). Moreover, no significant differences were observed between the MCT-ET group and either of the SAL groups, suggesting a restoration of these parameters.

### Oxidative stress and inflammation in gastrocnemius muscle: effect of combined ET

**Fig. 2:**
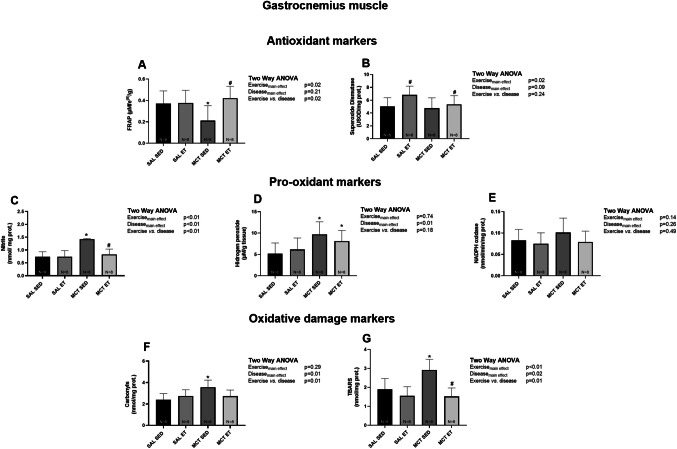
Effects of exercise training on oxidative stress markers in the gastrocnemius muscle. MCT: monocrotaline; SAL: saline; SED: sedentary; ET: exercise training. oxidative stress biomarkers. **A**, **B** Antioxidant markers (FRAP and SOD); **C–E** Pro-oxidant markers (nitrite, hydrogen peroxide, and NADPH oxidase); **F**,** G** Oxidative damage markers (carbonyls and TBARS). Data are expressed as mean ± SD. *p < 0.05 vs. SAL, #*p* < 0.05 vs. SED

Figure 2 illustrates the oxidative stress analysis in gastrocnemius muscle tissue. The MCT-SED group exhibited reduced FRAP levels compared to the SAL-SED group (*P* = 0.06; Cohen’s *d* = 1.42; Fig. 2A), indicating impaired antioxidant capacity. Combined ET restored FRAP levels in the MCT-ET group (P = 0.01; Cohen’s *d* = 1.89), bringing them to values comparable to the healthy controls. Combined ET also enhanced SOD activity in both the SAL and MCT groups (P_exercise_ = 0.02; η^2^ₚ = 0.20), improving antioxidant enzyme activity (Fig. 2B).

Pro-oxidant markers (Fig. 2C–E) showed increased nitrite levels in the MCT-SED group compared to the SAL-SED group (*P* < 0.01; Cohen’s *d* = 3.36; Panel C), while combined ET reduced nitrite levels in the MCT-ET group (*P* < 0.01; Cohen’s *d* = 2.90), restoring them to levels comparable to the SAL group. Regardless of the intervention, MCT groups exhibited higher hydrogen peroxide levels compared to the SAL groups (P_disease_ < 0.01; η^2^ₚ = 0.36; Fig. 2D), whereas NADPH oxidase activity showed no significant differences across the groups (Panel E).

Oxidative damage markers (Fig. 2F and G) showed increased protein oxidation (carbonyls) and TBARS in the MCT-SED group compared to the SAL-SED group (Cohen’s *d* = 2.16 and 1.91; carbonyls and TBARS, respectively). Conversely, combined ET significantly reduced carbonyl and TBARS levels (*P* < 0.01; Cohen’s *d* = 2.57) in the MCT-ET group, normalising them to levels observed in the SAL-SED group (Panels F and G).

Table 1 summarises the analysis of inflammatory mediators in the gastrocnemius muscle. Both PAH (P_disease_ = 0.04; η^2^ₚ = 0.05) and combined ET (P_exercise_ = 0.03; η^2^ₚ = 0.51) significantly reduced TNF-α levels. No significant effects of either PAH or ET were observed for IL-6 or IL-10 levels, suggesting that these interventions specifically modulate TNF-α without broadly affecting other inflammatory cytokines.

**Table 1:** Inflammatory markers obtained from the gastrocnemius and diaphragm muscles in trained and sedentary MCT and SAL groups

Inflammatory markers	MCT		SAL		ANOVA *P*-values		
	SED (*n*)	ET (*n*)	SED (*n*)	ET (*n*)	Disease	Exercise	Interac
Gastrocnemius							
Muscle weight (g)	1.12 ± 0.46* (7)	1.44 ± 0.25* (7)	1.52 ± 0.55 (6)	1.73 ± 0.30 (5)	0.04	0.12	0.74
IL-6 (pg/mg protein)	50 ± 14 (6)	43 ± 12 (9)	48 ± 23 (7)	42 ± 15 (7)	0.79	0.31	0.92
IL-10 (pg/mg protein)	12 ± 2 (5)	11 ± 2 (9)	12 ± 5 (7)	15 ± 6 (7)	0.22	0.88	0.23
TNF-α (pg/mg protein)	36 ± 7* (5)	28 ± 9*^#^ (9)	50 ± 16 (7)	35 ± 15^#^ (7)	0.04	0.03	0.49
Diaphragm							
Muscle weight (g)	0.88 ± 0.16* (7)	1.12 ± 0.36*^#^ (7)	1.00 ± 0.14 (6)	1.55 ± 0.45^#^ (4)	0.03	< 0.01	0.21
IL-6 (pg/mg protein)	30 ± 6* (5)	17 ± 5^#^ (8)	15 ± 1 (7)	15 ± 3 (7)	< 0.01	< 0.01	< 0.01
IL-10 (pg/mg protein)	1 ± 1* (6)	46 ± 7^#^ (7)	43 ± 1 (7)	36 ± 3 (7)	< 0.01	< 0.01	< 0.01
TNF-α (pg/mg protein)	45 ± 32* (6)	20 ± 8^#^ (9)	22 ± 5 (7)	24 ± 3 (8)	0.10	0.04	0.03

Data are presented as mean ± SD and were analyzed via two-way ANOVA, followed by the Tukey’s post hoc, when needed. MCT, pulmonary arterial hypertension induced by monocrotaline; SAL, healthy control group; Interac., interaction. *P < 0.05 *vs* SAL; #*P* < 0.05 *vs* SED

### Oxidative stress and inflammatory mediators in diaphragm muscle: effect of combined ET

**Fig. 3:**
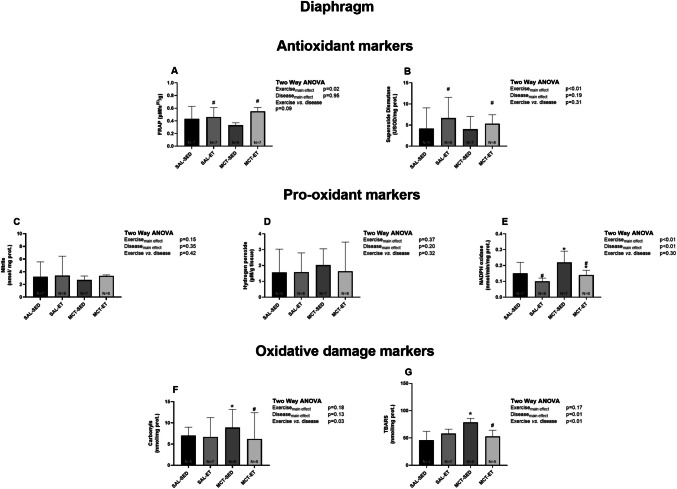
Effects of exercise training on oxidative stress markers in the diaphragm muscle. MCT: monocrotaline; SAL: saline; SED: sedentary; ET: exercise training. oxidative stress biomarkers. **A**,** B** Antioxidant markers (FRAP and SOD); **C–E** Pro-oxidant markers (nitrite, hydrogen peroxide, and NADPH oxidase); **F**,** G** Oxidative damage markers (carbonyls and TBARS). Data are expressed as mean ± SD. *p < 0.05 vs. SAL, #*p* < 0.05 vs. SED

Figure 3 illustrates the oxidative stress analysis in the diaphragm muscle. No differences were observed between the MCT and SAL groups for antioxidant markers (Fig. 3A and B); however, ET increased FRAP (P_exercise_ = 0.02; η^2^ₚ = 0.18) and SOD activity (P_exercise_ < 0.01; η^2^ₚ = 0.32) in both the SAL and MCT groups.

Regarding pro-oxidant parameters, no differences were found in nitrite or hydrogen peroxide levels (Fig. 3C and D, respectively). Regardless of the intervention, MCT groups exhibited higher NADPH oxidase compared to SAL (P_disease_ < 0.01; η^2^ₚ = 0.26; Fig. 3E). Conversely, ET reduced this pro-oxidant parameter in both MCT and SAL groups (P_exercise_ < 0.01; η^2^ₚ = 0.31).

Regarding oxidative damage, PAH increased carbonyl (P = 0.01; Cohen’s *d* = 1.03; Fig. 3F) and TBARS levels (*P* < 0.01; Cohen’s *d* = 2.22; Fig. 3G) compared to the SAL-SED group. Combined ET significantly reduced both carbonyl (P = 0.01; Cohen’s *d* = 1.01; Fig. 3F) and TBARS levels (*P* < 0.01; Cohen’s *d* = 1.86; Fig. 3G) in the MCT-ET group compared to MCT-SED, restoring these markers to levels comparable to those observed in SAL groups.

Table 1 summarises the analysis of inflammatory mediators in the diaphragm muscle. MCT-induced PAH significantly increased pro-inflammatory mediators (TNF-α and IL-6) and decreased the anti-inflammatory cytokine IL-10. Combined ET effectively prevented these alterations in the PAH. Specifically, when comparing the MCT-ET to the MCT-SED group, TNF-α (*P* = 0.02; Cohen’s *d* = 1.21), IL-6 (*P* < 0.01; Cohen’s *d* = 2.19), and IL-10 (*P* < 0.01; Cohen’s *d* = 5.10) levels were significantly improved, with values approaching those observed in SAL groups.

### Muscle weight and its association with oxidative stress and inflammatory mediators

The MCT groups exhibited significantly reduced gastrocnemius (P_disease_ = 0.04) and diaphragm (P_disease_ = 0.03) muscle weights compared to the SAL groups (Table 1). Although combined ET did not affect gastrocnemius muscle weight, it significantly increased diaphragm muscle weight across both groups (P_exercise_ < 0.01).

**Fig. 4:**
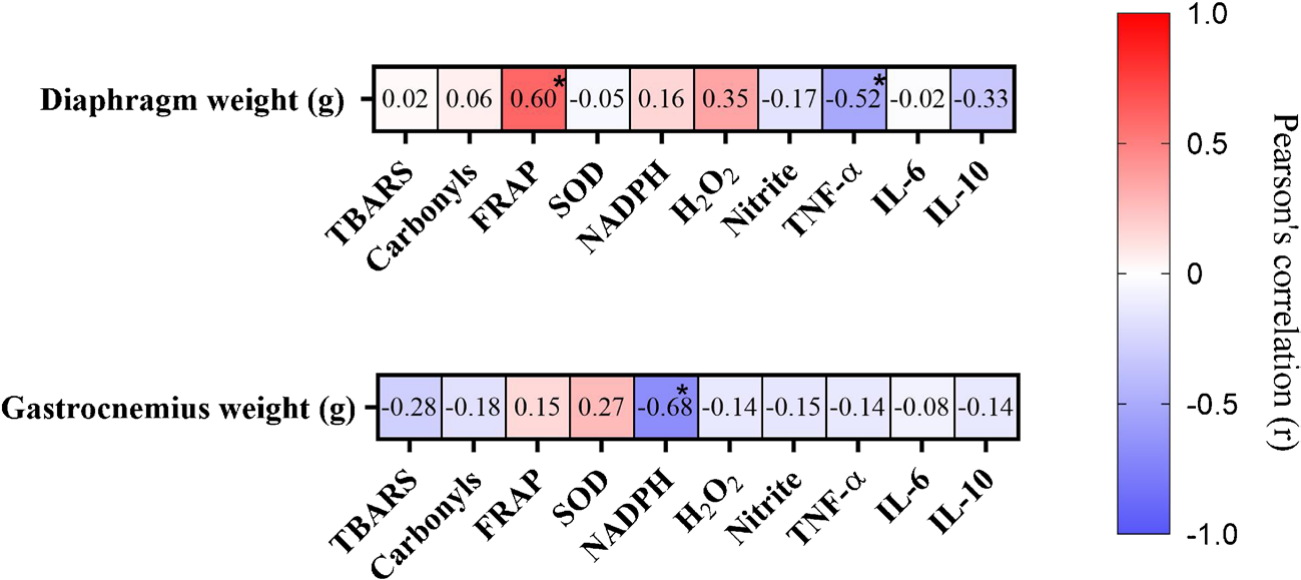
Correlation between oxidative stress, inflammation, and muscle weight in the diaphragm and gastrocnemius considering all groups. Warmer colours indicate positive correlations, while cooler colours indicate negative correlations. Data are expressed as Pearson’s correlation coefficient (r), with significance indicated (*p < 0.05)

The heatmap (Fig. 4) illustrates Pearson’s correlation coefficients (r) between diaphragm and gastrocnemius muscle weights and markers of oxidative stress and inflammation. For the gastrocnemius muscle, a strong negative correlation was observed with NADPH oxidase activity (r = –0.68, *P* < 0.05), suggesting that higher levels of this marker are associated with lower gastrocnemius muscle mass. For the diaphragm, a significant moderate positive correlation was found with FRAP (r = 0.60, *P* < 0.05) and a negative correlation with TNF-α (r = –0.52, *P* < 0.05). This suggests that higher antioxidant capacity is associated with greater diaphragm muscle mass, while increased inflammation, indicated by TNF-α levels, may contribute to muscle atrophy.

### Effect of combined exercise training on survival rate among PAH groups

**Fig. 5:**
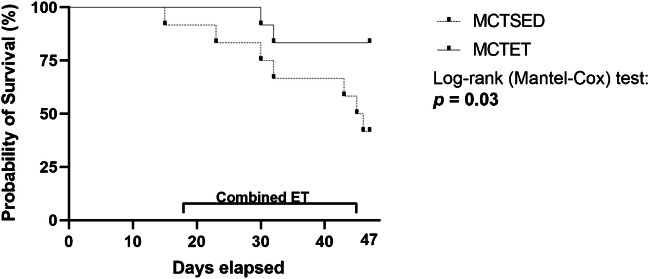
Survival rate of MCT-induced PAH rats with and without exercise training. Statistical analysis was performed using the log-rank (Mantel-Cox) test, *p* = 0.03

Combined ET significantly improved survival in PAH, with rates of 83% in the MCT-ET group compared to 41% in the MCT-SED group (Fig. 5; *P* = 0.03; hazard ratio = 4.3; 95% CI, 1.2–16.1).

## Discussion

This study evaluated whether a combined interval ET protocol, initiated after PAH onset, could improve oxidative stress and inflammation in the musculoskeletal system. We provide novel evidence that this exercise modality partially normalised oxidative stress and inflammation in both muscles and mitigated diaphragm muscle loss. These improvements were associated with reduced muscle wasting and enhanced survival in the PAH model.

### Musculoskeletal oxidative stress and inflammation in a preclinical model of PAH

Oxidative stress is a key contributor to pulmonary and right ventricular remodelling in the MCT-induced PAH model [43, 44, 60]. Elevated ROS and diminished antioxidant defences disrupt redox homeostasis, leading to endothelial dysfunction, vasoconstriction, and vascular remodelling [43, 44]. These imbalances, further exacerbated by hypoxia and inflammatory mediators, contribute to structural lung changes and impaired pulmonary function, ultimately driving PAH progression. Moreover, it is increasingly recognized that the ROS and inflammation profile extends beyond the pulmonary system to affect the musculoskeletal system [53, 56]. In line with this, our findings demonstrate significant alterations in the gastrocnemius muscle, a key peripheral muscle impacted by PAH.

Specifically, we observed reduced antioxidant defences (e.g., FRAP) and increased pro-oxidant activity (e.g., elevated hydrogen peroxide levels) in PAH compared to healthy controls. The PAH-SED group also exhibited higher nitrite levels in the gastrocnemius, consistent with previous findings in MCT-induced PAH [39]. This increase may reflect a compensatory role of nitric oxide (NO) in counteracting excessive ROS-mediated vasoconstriction [39, 58]. Nitrite, a stable metabolite of NO, serves as an indirect marker of endothelial NO production. However, excessive NO levels can lead to peroxynitrite (ONOO⁻) formation, a highly reactive nitrogen species that contributes to muscle oxidative damage, including protein carbonylation and lipid peroxidation (e.g., TBARS) [58].

Interestingly, TNF-α levels were lower in the gastrocnemius of MCT-induced PAH animals compared to controls. This reduction may represent an initial compensatory response to elevated ROS levels in this muscle, independent of exercise [2].

The PAH, regardless of exercise, presented with reduced gastrocnemius muscle mass compared to healthy controls. This indirect evidence of muscle wasting strongly correlated with increased NADPH oxidase activity. These findings align with broader evidence indicating that oxidative stress and inflammation contribute to peripheral muscle atrophy and diminished functional capacity in chronic diseases such as PAH [41, 43, 49, 56].

Our study also revealed significant oxidative stress and inflammatory alterations in the diaphragm, a critical respiratory muscle. We observed increased NADPH oxidase activity, elevated lipid peroxidation (e.g., TBARS), and heightened inflammatory markers (e.g., IL-6 and TNF-α). These findings align with evidence highlighting the diaphragm’s particular vulnerability to PAH-induced damage [28, 53]. For instance, oxidative stress-driven activation of proteolytic pathways, such as the ubiquitin–proteasome system, along with mitochondrial dysfunction, are key mechanisms linking ROS and inflammation to diaphragm muscle weakness [42, 53]. IL-6, in particular, has been implicated in skeletal muscle atrophy in preclinical disease models [60, 70], a finding corroborated by our observation of increased IL-6 levels in the diaphragm.

Our findings also demonstrated that reduced antioxidant activity (e.g., FRAP) and elevated inflammatory markers (e.g., TNF-α) were moderately associated with muscle loss. This is particularly relevant, as diaphragm atrophy not only exacerbates respiratory dysfunction [52] but is also linked to worse clinical outcomes, including reduced survival and exercise tolerance in chronic diseases like PAH [49, 59].

Together, our results reinforce the systemic impact of PAH, extending beyond the pulmonary vasculature and heart to skeletal muscles. Oxidative stress and inflammation play pivotal roles in driving muscle atrophy and dysfunction, underscoring the importance of targeting these mechanisms in PAH treatment strategies.

### The protective effects of combined exercise training

Combined ET has been proposed as a non-pharmacological intervention to mitigate the systemic and muscle-specific complications of PAH. However, evidence on impact of this modality on ROS and inflammatory markers are still lacking. Recent findings by Leite et al*.* [44, 45] provided the first evidence that combined ET mitigates oxidative stress in the lungs and right ventricle, reducing pulmonary and cardiac remodeling in PAH. These effects were accompanied by improvements in echocardiographic markers of pulmonary vascular resistance. While these latter results were also observed in our study, our findings extend this evidence by demonstrating, for the first time, that combined ET also modulates musculoskeletal oxidative stress and inflammatory markers in a preclinical PAH model. In addition to reinforcing the cardiovascular benefits of ET, our findings highlight its potential to counteract the systemic impact of PAH on skeletal muscle, a previously overlooked aspect of the disease.

Specifically, combined ET restored antioxidant defences (e.g., increased SOD activity, normalised FRAP levels), reduced pro-oxidant markers (e.g., hydrogen peroxide, nitrite), and attenuated oxidative damage (e.g., decreased TBARS and protein carbonyls) in both the gastrocnemius and diaphragm. These findings not only confirm the benefits of ET in mitigating oxidative stress but also underscore its role in counteracting systemic complications in PAH, such as muscle wasting. Notably, Koichi et al. [37] demonstrated that a mimetic of SOD and CAT prevented diaphragm muscle weakness in MCT-induced PAH model, further supporting the critical role of enhanced antioxidant activity in preserving muscle function. The ability of ET to increase SOD and CAT activity may similarly contribute to improved diaphragm muscle function by reducing oxidative damage and maintaining contractile properties, which are essential for respiratory efficiency and overall clinical outcomes in PAH [28, 60].

Combined ET also shifted the inflammatory profile toward an anti-inflammatory state, reducing pro-inflammatory cytokines (e.g., TNF-α, IL-6) and restoring IL-10 levels in the diaphragm. IL-10 is particularly relevant under conditions of elevated oxidative stress, as it has been shown to improve SOD and CAT activities [27], further enhancing antioxidant defences. Notably, ET prevented diaphragm muscle wasting, a key factor in improving respiratory function and overall prognosis in PAH [5]. This protection against muscle atrophy underscores the importance of targeting oxidative stress and inflammation-induced catabolic pathways through interventions like ET.

Combined ET improves oxidative stress and inflammatory profiles through multiple mechanisms. In addition to its direct effects on the inflammatory cascade, ET enhances mitochondrial function, a key determinant of cellular redox balance. As mitochondria are a major source of ROS and particularly vulnerable to oxidative damage, their dysfunction can exacerbate ROS production, creating a vicious cycle of oxidative stress [14, 24, 40, 60, 70]. Combined ET also improves autonomic modulation [33], which can favorably influence the inflammatory process and redox balance [23]. Additionally, ET improves skeletal muscle blood flow [33], which is particularly relevant as hypoperfusion is known to induce inflammatory responses and increase ROS production in PAH [28, 50].

### Clinical relevance: improved muscle weight and survival

The systemic benefits of ET extend beyond improvements in redox balance and inflammation. Our study demonstrated that ET enhances survival in MCT-induced PAH, which might be partially due to its protective effects against muscle wasting and systemic complications. Indeed, in chronic conditions such as heart failure and chronic obstructive pulmonary disease, diaphragm weakness and atrophy—mediated by increased ROS production—contribute to respiratory failure and increased mortality risk. Conversely, ET itself mitigated diaphragm atrophy and improved the ROS profile, highlighting its therapeutic potential. These benefits were achieved using a multimodal ET protocol that combined aerobic and resistance training, a widely adopted approach in rehabilitation centers due to its safety and clinical relevance [33, 35].

This study employed moderate-intensity interval training, tailored to PAH severity, to replicate intensities commonly used in rehabilitation settings [32, 35]. Interval training offers a safe yet effective approach, whilst providing significant systemic and muscle-specific benefits [13, 20]. Additionally, it has been shown to outperform continuous exercise training in PAH, with studies demonstrating improvements in right ventricular function, exercise capacity, and peripheral adaptations [13, 20].

### Methodological considerations and limitations

The MCT-induced PAH model is widely recognised as a robust and reproducible method for studying the pathophysiology of PAH and testing therapeutic interventions [28, 36, 44, 45]. Its widespread use is attributed to its simplicity and reliability in inducing pulmonary vascular remodelling, increased pulmonary vascular resistance, and elevated mPAP, closely mirroring the clinical manifestations of human PAH [31]. A single dose of 40 mg/kg monocrotaline was used based on well-established evidence, ensuring stable pulmonary hypertension with preserved cardiac output [36].

In our study, we confirmed the validity of this model through echocardiographic indicators, including reduced PAAT/EjT – a validated index of increased PVR – increased mPAP and PA/Ao ratio, markers strongly correlated with mortality [65, 67]. Additionally, the dagger-shaped Doppler waveform observed in echocardiography further supports the presence of significant haemodynamic alterations [67].

Although invasive haemodynamic measurements are considered the gold standard for confirming PAH, our findings are well-supported by established echocardiographic parameters [67], which are non-invasive and clinically relevant. Importantly, these echocardiographic markers were responsive to our ET intervention, further validating the model’s applicability in assessing therapeutic efficacy. Overall, the MCT-induced PAH model provided a reliable platform for exploring the systemic and muscle-specific effects of PAH and evaluating the benefits of ET.

Despite its promising findings, this study has limitations. First, the intervention duration was constrained by the MCT-induced PAH time course, with four weeks being optimal to elicit benefits [44, 45]​ prior the progression of the disease [36]. This short period may limit the generalisability of long-term adaptations. Second, only male rats were used, as females exhibit greater resistance to MCT-induced PAH, affecting disease progression and successful induction within a defined timeframe [7]. This limits the applicability of our findings, highlighting the need for future studies on sex-specific responses to ET in PAH.

## Conclusion

Using a multimodal ET protocol that combines aerobic and resistance training, commonly used in rehabilitation, this study demonstrated improvements in redox balance and inflammatory profiles in both peripheral and respiratory muscles. These adaptations were linked to reduced diaphragm muscle wasting and enhanced survival. Our findings support combined interval ET as a viable non-pharmacological strategy for managing systemic complications of PAH.

## Data Availability

The datasets generated and/or analysed during the current study are available from the corresponding author on reasonable request.
